# Tunnel Cross-Section Deformation Monitoring Based on Mobile Laser Scanning Point Cloud

**DOI:** 10.3390/s24227192

**Published:** 2024-11-09

**Authors:** Mahamadou Camara, Liying Wang, Ze You

**Affiliations:** School of Geomatics, Liaoning Technical University, Fuxin 123000, China; mahamadoucamaramoc@outlook.com (M.C.); youze1997@163.com (Z.Y.)

**Keywords:** MLS, point cloud, tunnel cross-section, deformation monitoring, data management, ellipse fitting

## Abstract

Mobile laser scanning (MLS) has emerged as a pivotal tool for accurately collecting tunnel point cloud data and enabling the detection of tunnel deformation. This study introduces a novel approach for the precise monitoring of tunnel cross-section deformation, a critical factor in assessing stability and lining safety. The MLS system used in this study is the Self-mobile Intelligent Laser Scanning System (SILSS) for data acquisition. A comparison with corresponding data acquired by Leica P16 demonstrates that the data collected by SILSS are accurate. The methodology developed utilizes ellipticity parameters and deformation analysis indices based on the ellipse-fitting analysis of circular shield tunnel deformation. A key innovation is the robust denoising of data using the Random Sample Consensus (RANSAC) method, ensuring accurate ellipse fitting and extraction of tunnel lining. Subsequently, an algorithm segmented the tunnel cross-section lining into individual shield tunnels, enabling the calculation of ellipticity parameters for shield tunnels, which are the objects for deformation analysis. The experimental results underscore the novelty and effectiveness of this approach in monitoring deformation across different indices. The method proves to be a reliable tool for assessing tunnel health, providing a detailed evaluation of the cross-section’s condition through statistical and graphical visualization. This study significantly advances shield tunnel monitoring, offering a practical and precise methodology for tunnel deformation analysis based on MLS point cloud data.

## 1. Introduction

Shield tunnels, a staple in urban subway construction, are crucial for urban infrastructure due to their stability and ease of installation. However, these tunnels are susceptible to issues such as water leakage, collapse, and deformation, which can compromise their structural integrity and safety [1]. These issues become more pronounced after approximately 10 years of operation as many tunnels start to exhibit signs of aging and structural degradation due to material wear, changing geological conditions, and extended service life. Therefore, continuous shield tunnel structure deformation monitoring is vital for ensuring public safety. Traditional methods of tunnel deformation monitoring, which rely on manual inspections and stationary sensors like inclinometers and strain gauges, have significant limitations. These methods, still in use, employ total stations, Leica, and convergence instruments. However, they often suffer inefficiencies, lower accuracy, and incomplete spatial coverage. Manual inspections, in particular, are time-consuming and prone to human error, often providing only fragmented views of a tunnel’s overall condition [2,3,4,5]. Conventional sensor-based methods, while helpful, are constrained by their fixed installations, which fail to capture the complete scope of tunnel deformations. Due to the limitation of the number of monitoring points, these traditional methods can only measure the deformation of a very limited set of points, resulting in insufficient monitoring precision. They do not provide a complete tunnel wall model [6].

The need for more advanced and comprehensive methods to monitor tunnel safety has become apparent, necessitating the development of surveying and mapping technology, which has significantly transformed conventional measurement methods. The rapid advancement of three-dimensional (3D) laser scanning and vehicle mobile measurement systems has introduced new methods characterized by high precision, speed, and non-contact capabilities [7]. Mobile 3D laser scanning technology is now widely used in various fields, such as street view scanning [8], urban road extraction [9], building modeling, infrastructure management, and mobility evaluation [10,11]. Due to its efficiency, quality, and resistance to environmental influences, mobile laser scanning (MLS) technology has proven especially useful in tunnel measurements. MLS technology has emerged as a game-changer for tunnel deformation monitoring. Mounted on mobile platforms, MLS systems use high-precision laser scanners to rapidly and comprehensively acquire 3D point cloud data of tunnel interiors [12,13]. MLS offers significant advantages over traditional methods by enabling the precise detection of deformations and providing comprehensive spatial coverage. It has proven to be a cost-effective solution for monitoring unstable tunnel surfaces [14]. Recent studies have shown that MLS technology is highly effective for tunnel deformation monitoring, capturing critical data that aid in maintenance and safety strategies. For instance, Wu et al. [15] used 3D laser scanning to monitor long- and short-term tunnel deformation at key points during different excavation stages. Zhang et al. [16] proposed a mobile 3D laser measurement scheme for tunnel monitoring, reflecting the growing use of laser scanning in deformation analysis [3], point-cloud denoising [17], and tunnel section extraction [18]. Van et al. [19] mapped tunnel point clouds onto a cylindrical surface for deformation measurement, while Zhu [20] used cross-section intercepts of point clouds to fit ellipses for monitoring. Lindenbergh et al. [21] demonstrated higher accuracy using circle-fitting techniques with a panoramic laser scanner, though some deformation sections were not elliptical. Walton et al. [22] and Li et al. [23] improved deformation analysis with elliptical fitting, and Xie et al. [24] developed a point-cloud processing algorithm validated by field experiments. Hu et al. [25] employed the Kriging filtering algorithm to process point-cloud data, verifying its accuracy against traditional methods.

Despite its advantages, MLS data processing presents its own set of challenges. The presence of noise and outliers in the point clouds can reduce the accuracy of analyses and visualizations [26,27,28]. Therefore, refining MLS data processing techniques is crucial for reliable and accurate deformation assessments. Accurate point cloud data are essential for tunnel deformation monitoring, especially when utilizing ellipse-fitting methods to evaluate tunnel structural stability. Overcoming these challenges requires the implementation of advanced noise and outlier removal techniques. Several methods have been developed to enhance point cloud data quality, including PointCVaR optimization and deep learning-based pipelines like Re-VISOR [29,30]. Additionally, filtering methods such as cloth simulation and the AORI filter have been proposed to refine data accuracy by removing artifacts [31,32]. Techniques such as cylindrical projection and 3D laser point cloud positioning are now widely used for tunnel extraction and deformation analysis [18,28]. These techniques help identify critical structural features like waist seams, which are vital for accurate deformation analysis [33,34]. Semantic segmentation, Kriging filtering algorithms, and deep learning methods have also been employed to improve the precision of tunnel component detection and real-time deformation analysis [35,36]. Compared with common methods, the MLS point cloud can easily acquire high-density and high-precision observation data with high sampling frequency, which can make effective measurements of complex curved tunnel structures. As a result, it receives special attention in terms of tunnel deformation control and monitoring. Currently, the emphasis is placed on topics such as deformation tracking, the deformation algorithm of subway tunnels, and deformation characteristics of tunnel cross-sections.

This study employed the Mobile Laser Scanning (MLS) technique for precise tunnel cross-section deformation monitoring, capitalizing on the high precision and density of MLS point clouds. The method focuses on extracting tunnel structures and analyzing cross-sections over two measurement phases. After acquiring the point cloud data, key steps were performed, including non-lining point removal, shield ring separation, and ellipticity parameter extraction using the ellipse-fitting method. To assess deformation, the ellipticity parameters from each shield ring were compared with those of the tunnel design. Additionally, ellipticity, center displacement, and axis length change were computed for the shield rings in the test area. Radial deformation was calculated by angle using the ellipse-fitting method. These metrics were then used to evaluate the local and overall health of the shield tunnel. The difference between the data measured and the designed cross-section was analyzed and compared with the corresponding data acquired by the Leica P16 system for accuracy validation of this method.

## 2. Data Acquisition System

**Figure 1:**
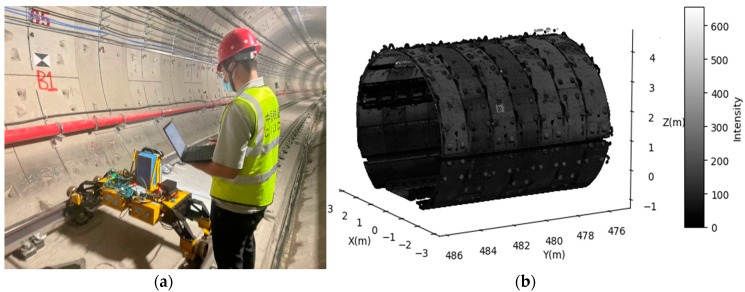
Tunnel point cloud data collection system. (**a**) MLS System; (**b**) tunnel cross-section point cloud.

The test was conducted on the Guangzhou Metro Line 2, a circular shield tunnel with an inner diameter of 5.4 m and a ring width of 1.2 m. The absolute elevation of the tunnel ranges from −9.90 m to −10.45 m. This system efficiently recorded tunnel cross-sections through high-resolution point clouds. The rail-borne MLS system used in this study, the Self-mobile Intelligent Laser Scanning System (SILSS), was independently developed by China Railway Design Group Co., Ltd., located in Tianjin, China for data acquisition. As shown in Figure 1, the system is mounted on a rail vehicle, with an operator monitoring the process via a laptop. It allowed scanning at speeds of 1 km/h, achieving a point density of 100 lines per second. The SILSS system includes several components: a high-precision laser scanner that captures 360-degree profiles, a control unit that synchronizes scanning with rail vehicle movement, a positioning system that tracks the rail vehicle’s location and orientation, and a data-storage unit for recording the point cloud data. The laser scanner operates at a resolution of 100 profiles per second, capturing tunnel cross-sections with a profile interval of 5.6 mm and a laser point spacing of 1.6 mm. The scanner moves at 2 km/h (0.56 m/s), ensuring precise profiling of the tunnel’s geometry, which is essential for detecting deformations. This system was used to collect the point cloud data(t) of the 17 cross-sections as our test survey area, which contains 104 shield rings. Figure 1 illustrates the MLS used during this data acquisition (Figure 1a) and a cross-section point cloud sample measured using MLS, shown in Figure 1b.

After capturing the point cloud data using the MLS, python 3.10 programming through a Jupyter notebook 7.2.1 was employed to denoise MLS point cloud data. Then, single shield rings and ellipticity parameters were extracted, and tunnel cross-section deformation was detected based on the ellipse-fitting method. Finally, the point cloud data from the Leica system were compared to SILSS data for this method validation.

## 3. Methods and Materials

**Figure 2:**
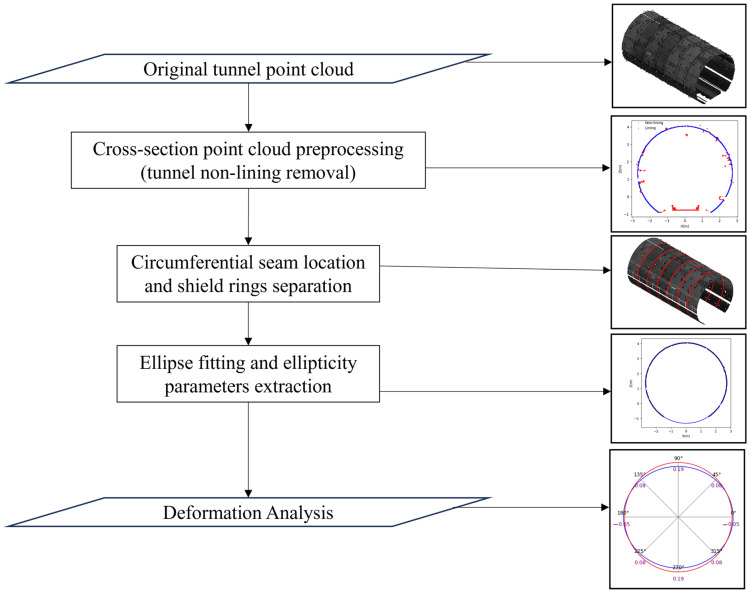
Flow chart of the method proposed.

This study used the tunnel cross-section MLS point cloud for deformation analysis. Both data are measured from the same cross-sections. The method employed in this study begins with cross-section point cloud data acquisition, tunnel non-lining removal, individual shield ring separation, ellipse fitting, ellipticity parameters extraction, and deformation analysis based on the deformation metrics indices. These processes were executed on both data separately for accurate validation. Then, the ellipticity parameters were used to define each shield ring’s ellipticity change, ellipse center displacement, axis change, and health degree analysis and visualize the tunnel cross-section radial deformation by the angle from 0° to 360°. This deformation analysis was conducted in two phases: the shield tunnel design point cloud data and the SILSS data measured used for deformation analysis and comparison with data from the Leica method for this method validation. The overall workflow for the method is illustrated in Figure 2.

### 3.1. Cross-Section MLS Point Cloud Data Preprocessing

This step involved cleaning the data point clouds measured and removing cross-section non-lining. The clean point cloud data for each cross-section of our shield tunnel were extracted. Subsequently, circumferential seams were identified, and the individual shield ring was separated as the research object. This is an important step in the tunnel deformation monitoring process.

#### 3.1.1. Non-Lining Points Removal

**Figure 3:**
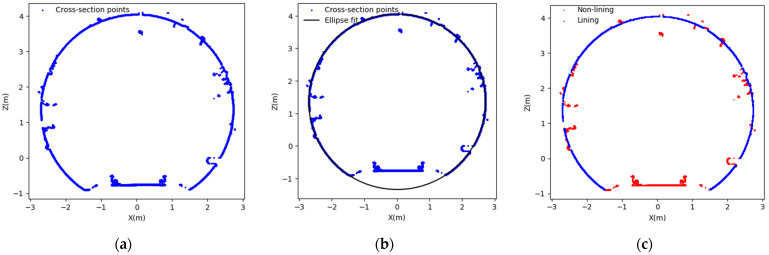
Illustration of the tunnel cross-section non-lining points cloud removal process, 2D view. (**a**) Survey tunnel cross-section; (**b**) ellipse fit the cross-section point cloud; (**c**) non-lining points of the tunnel cross-section, shown in red.

Given that the shield tunnel shape can be approximated as an ellipse close to a standard circle [37,38,39], the proposed method utilizes an iterative approach to fit an ellipse, effectively excluding noise points, as illustrated in Figure 3. The process begins by selecting the point cloud within each dataset, removing the lower part (which contains more tunnel facilities), and focusing on the upper part (which contains fewer or no non-lining points). The upper section of each cross-section is selected, as it contains fewer non-lining points compared to the lower section, which often includes tunnel facilities. The selected point clouds are then processed using the Random Sample Consensus (RANSAC) algorithm to fit a 3D circle. The residuals between the fitted circle and each point in the cross-section point cloud are then computed. If a residual value is less than zero or exceeds a specified threshold (Td), the corresponding point is classified as noise and removed. If the residual falls within the threshold, the point is retained as a tunnel lining point. The threshold (Td) is calculated based on the statistical characteristics of the residuals between the fitted 3D circle and the tunnel cross-section point cloud. Td is calculated by the residuals’ mean and standard deviation after the circle is fitted. It is set as a multiple of the standard deviation, such as Td=μ+kσ, where μ is the mean of the residuals, σ is the standard deviation of the residuals, and k is a chosen multiplier (3) based on the desired strictness for outlier detection. This value is fine-tuned and used to provide a benchmark for accurately identifying tunnel lining points. This tuning process enhances the accuracy of the fitted circle and ensures the reliable detection of the tunnel’s lining structure.

Overall, this methodology provides a robust approach for preprocessing tunnel cross-sectional data by effectively removing noise and fitting geometric shapes to the cleaned data. Using RANSAC ensures that the model fitting is resistant to outliers, while ellipse fitting accurately captures the geometric properties of the tunnel’s cross-sections [40]. This process is essential for reliable deformation monitoring and data analysis.

**Figure 4:**
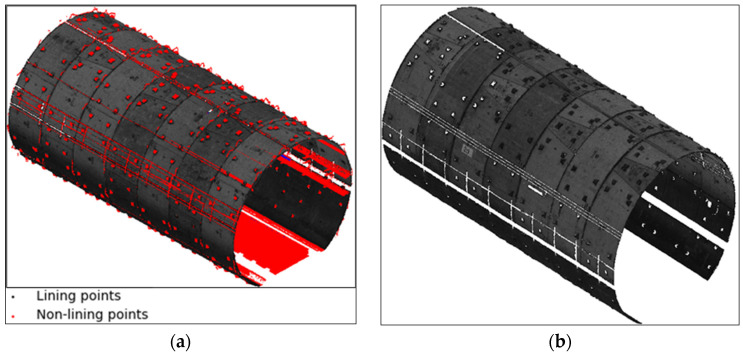
Non-lining points removal using the proposed non-lining points removal algorithm 3D view. (**a**) Comparison of the cross-section non-lining points removed and cross-section lining; (**b**) tunnel cross-section lining point clouds.

The mobile measurement technique generates a point cloud representing the tunnel structure, including the outlier and noise. Preprocessing was essential for removing noise and outliers, and this was achieved using a robust cross-section lining point cloud extraction algorithm. The RANSAC algorithm was applied to identify and remove outliers from the raw point cloud. This process involves fitting an ellipse to each tunnel cross-section. The threshold is set to be slightly larger than the expected segment joint depth so that points near joints, which naturally deviate from the fitted ellipse, are not mistakenly removed as noise. This ensures that only points significantly deviating from the tunnel’s geometry are identified as outliers. The RANSAC algorithm can more accurately differentiate between genuine structural points and noise by considering these geometric properties. Once the outliers are removed, the remaining tunnel cross-section points, as shown in Figure 4, are used for further analysis in the study.

#### 3.1.2. Circumferential Seam Location and Shield Ring Separation

Denoising was followed by individual shield ring separation by extracting the circumferential seam via intensity values. The mobile laser scanner assigns an intensity value to each point in a point cloud captured. Higher-intensity values correspond to stronger reflections of the object, while lower values indicate weaker reflections.

Due to the difference in material, color, and surface roughness of the tunnel’s inner wall, the laser reflection intensity between the ring and ring joint (seam) varies. The average intensities of the ring circumferential joint are not evenly distributed. Still, all their values are significantly lower than that of the segment on both sides, showing as local minimums. From this, an adaptive multiple-threshold algorithm based on the local intensity statistics is proposed to extract the circumferential joint.

**Figure 5:**
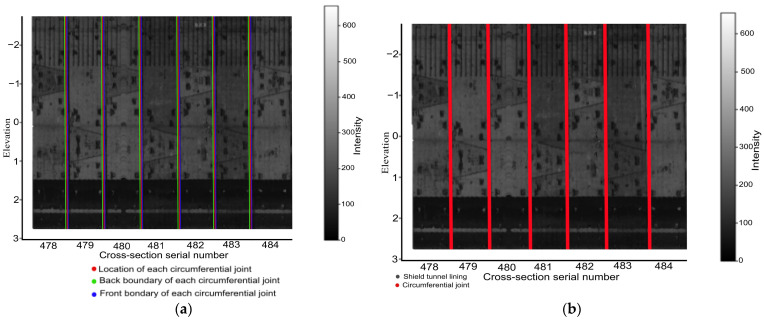
Illustration of the shield tunnel separation. (**a**) Location of circumferential joints (seams); (**b**) individual shield ring identified.

Rings corresponding to local minima are identified using valley detection. First, the average intensity of each cross-section is calculated, creating a feature set m_k_. Next, the principle of valley detection is applied to locate tunnel cross-sections that correspond to local minima, following Equation (1). Specifically, suppose *m_k_* is the local minimum compared to its *N* adjacent shield ring on both the front and back, and the difference between *m_k_* and the average intensity of the *N* preceding (or succeeding) ring exceeds a threshold (*T*). In that case, *m_k_* is identified as a valley value. The corresponding ring is then located and used as the initial position of a circumferential joint. These detected shield rings are shown in Figure 5b.

(1) $$\left\{\begin{array}{c} m_{\min}^{\left(k\right)}=\min\left\{m_{k-N},\cdots ,m_{k},\cdots ,m_{k+N}\right\}\\ \left|m_{\min}^{\left(k\right)}-\frac{1}{N}\sum_{k^{\prime }=k-2N}^{k^{\prime }=k-1-N}m_{k^{\prime }}\right|\geq T\\ \left|m_{\min}^{\left(k\right)}-\frac{1}{N}\sum_{k^{\prime }=k+1+N}^{k^{\prime }=k+2N}m_{k^{\prime }}\right|\geq T \end{array}\right.$$

The threshold *T* is determined using a statistical analysis method T=M−a⋅S, where a is a multiplier set to 3, based on the 3σ-rule. *M* and *S* represent the mean and standard deviation of the feature set *m_k_*, respectively $M=\frac{\sum_{k=1}^{q}m_{k}}{q}$, $S=\sqrt{\frac{\sum_{k=1}^{q}\left(m_{k}-M\right)}{q}}$. The adjacency size *N* is optimized according to the width of the circumferential joints in the original point clouds.

When determining the boundaries and location of each circumferential joint, as shown in Figure 5b, circumferential joints in the tunnel cross-section have a certain width. It is necessary to accurately determine each circumferential joint’s front and back boundaries for precise identification. To account for the intensity variations of the circumferential joint and the segments on both sides, the mean $\mu_{j}^{L}$ ($\mu_{j}^{R}$) and standard deviation $\sigma_{j}^{L}$ ($\sigma_{j}^{R}$) of the N shield tunnel on the front and back sides of the circumferential joint were first calculated using Equation (2). For the shield ring within $c_{j}-N,c_{j}$, the first ring with an average intensity less than $\mu_{j}^{L}-b\cdot \sigma_{j}^{L}$ was identified and marked as $c_{j}^{L}$ (illustrated by the green line in Figure 5a. Similarly, for shield rings within $c_{j},c_{j}+N$, the last ring with an average intensity less than $\mu_{j}^{R}-b\cdot \sigma_{j}^{R}$ was identified and marked as $c_{j}^{R}$. The multiplier *b* is set to 3, following the 3σ-rule in the experiment. Finally, the shield rings within $c_{j}^{L}$ and $c_{j}^{R}$ were detected, such as in Figure 5. The average of $c_{j}^{L}$ and $c_{j}^{R}$ is used as the location information for the circumferential joint.

(2) $$\mu_{j}^{L}=\frac{\sum_{u=c_{j}-2N}^{c_{j}-N-1}m_{u}}{N}\mu_{j}^{R}=\frac{\sum_{u=c_{j}+N+1}^{c_{j}+2N}m_{u}}{N}\sigma_{j}^{L}=\sqrt{\frac{\sum_{u=c_{j}-2N}^{c_{j}-N-1}\left(m_{u}-\sigma_{j}^{L}\right)}{N}}\sigma_{j}^{R}=\sqrt{\frac{\sum_{u=c_{j}+N+1}^{c_{j}+2N}\left(m_{u}-\sigma_{j}^{R}\right)}{N}}$$

### 3.2. Shield Rings Ellipticity Processing

A shield ring comprises several precast segments that encase the tunnel’s periphery completely circularly when laid in proper sequence. A shield tunnel is further formed by connecting several of these shield rings in the end-to-end form, giving a continuous concrete tunnel lining with improved structural integrity. Following the previous ring seam identification, the point cloud of each shield ring was projected to the XZ plane. The *X*-axis represents the horizontal coordinate along the tunnel’s length, and the *Z*-axis represents the vertical changes, such as the tunnel’s height or elevation profile. Obtained 2D point cloud sets were fitted using the RANSAC elliptic fitting algorithm.

After projecting the shield ring point cloud into 2D, an ellipticity parameter algorithm was used to fit the ellipse to each shield ring point cloud and extract ellipse parameters, as demonstrated in Table 1 and Table 2 for further analysis as deformation through key metrics.

Ellipticity is a valuable metric for assessing the shape of objects and is crucial in tunnel deformation monitoring. It quantifies the distortion of an ellipse shape, aiding decision-making [41]. The choice of the ellipticity formula depends on the context and goals, with various methods available [42]. The ellipticity ratio provides a straightforward measure for shield tunnels, though more detailed assessments may benefit from using eccentricity or its square. The cartesian coordinate system is utilized throughout this procedure, and the angle theta (θ) was adjusted to rotate the ellipse during fitting. The data were presented in a cartesian coordinate system, where x and z were represented by the point coordinates. The key parameters above were obtained from the ellipse-fitting process for deformation analysis. First, the centers $\left(c_{x},c_{y}\right)$ of the ellipses were specified within the x and z coordinate plane, where $c_{x}$ was the x coordinate and $c_{y}$ was the z coordinate of the ellipse’s center. Then, the semi-major axis (a) was represented by half of the ellipse’s longest diameter, while the semi-minor axis (b) was half of the shortest diameter. The rotation angles (θ) of the ellipse’s orientation relative to the coordinate axes were also described. These parameters collectively define the ellipse’s position, size, and orientation, which were crucial for accurately modeling the shield tunnels and analyzing any deformations. The ellipticity (e) of the different shield rings was calculated based on Equation (3) for ellipticity change detection.

(3) $$e=\sqrt{1-{\left(\frac{b}{a}\right)}^{2}}$$

The ellipse-fitting algorithm minimizes the error defined by the **ellipse_error** function, which calculated the difference between the data points and the ideal ellipse equation: $\frac{x^{2}}{a^{2}}+\frac{y^{2}}{b^{2}}=1$. This error function computed the distance of each point from satisfying the general ellipse Equation (4).

(4) $${\left(\frac{\left(x-c_{x}\right)\cos\left(\theta \right)+\left(y-c_{y}\right)\sin\left(\theta \right)}{a}\right)}^{2}+{\left(\frac{\left(x-c_{x}\right)\sin\left(\theta \right)-\left(y-c_{y}\right)\cos\left(\theta \right)}{a}\right)}^{2}-1=0$$

The error function evaluated how much each data point deviated from this ideal ellipse, aiming to minimize the sum of squared residuals. This algorithm iteratively adjusts the ellipse parameters ($c_{x}$, $c_{y}$, a, b, and θ) to achieve the best fit by minimizing the discrepancies between the observed data points and the estimated ellipse. Combining the robustness of gradient descent with the efficiency of the Gauss–Newton method, the algorithm effectively refined the parameters to provide an optimal fit for the given data. This approach effectively captures the underlying shape of the tunnel’s shield rings, which are crucial for applications such as tunnel deformation monitoring.

Shield ring ellipticity processing is important in this tunnel deformation analysis. It involved extracting key parameters for deformation analysis based on the ellipse-fitting method. This process was executed on 104 shield rings, representing our test area of the shield tunnel for two different measurements, as shown in Table 1 based on SILSS using the MLS system and validated by ellipticity parameters in Table 2 extracted through point cloud using the Leica P16 system. Those data extracted from the ellipticity parameters analysis based on MLS point clouds are compared to tunnel design formation analysis for each period.

### 3.3. Deformation Detection

In this part, we present the methodology for monitoring the shield tunnels’ deformation using an ellipse-fitting technique. This approach leverages data from MLS point clouds measured to analyze the shield tunnel deformation and identify deviations from their intended design. The process starts with tunnel design point cloud processing to extract ellipticity parameters. Based on the MLS data from the ellipticity processing section, the deformation was analyzed for each shield ring.

Given the parameters of ellipses, various deformation metrics were computed, including ellipticity change ΔE, center displacement of the ellipse centers ΔC, axis length changes $\Delta_{ab}$, and angle orientation change Δθ. To quantitatively assess the structural health degree of the tunnel, these key metrics were calculated accordingly, and radial deformation at multiple angles ΔR. These metrics were computed using the following equations:

Ellipticity change is defined as the difference in the shape of an ellipse, which is essential to quantify the circular shield tunnel deformation. It is calculated using Equation (5).

(5) $$\Delta E=E_{measured}-E_{design}$$

where $E_{measured}$ is the ellipticity of the measured ellipse and $E_{design}$ is the ellipticity of the design ellipse.

The center displacement of the ellipse is given by the distance between the centers of the two ellipses. The Euclidean distance between the centers of the ellipses (ellipse design and ellipse measured) is computed using Equation (6).

(6) $$\Delta C=\sqrt{{\left(c_{xi}-c_{x0}\right)}^{2}+{\left(c_{yi}-c_{y0}\right)}^{2}}$$

where $\left(c_{x0},c_{y0}\right)$ are the coordinates of the center of the shield ring design and $\left(c_{xi},c_{yi}\right)$ represents the coordinates of the center of the shield ring measured.

The axis length change is calculated as shown in Equation (7).

(7) $$\Delta_{ab}=\left|a_{i}\right.-\left.a_{0}\right|+\left|b_{i}\right.-\left.b_{0}\right|$$

where $a_{0}$ and $b_{0}$ are the semi-major and semi-minor axes of the ellipse design, and $a_{i}$ and $b_{i}$ are the semi-major and semi-minor axes of the ellipse measured.

The change in orientation angle is given by Equation (8). It is calculated for further analysis, such as the shield ring health score $\Delta_{hs}$.

(8) $$\Delta \theta =\left|\theta_{i}\right.-\left.\theta_{0}\right|$$

where $\theta_{0}$ is the orientation angle of the design ellipse and $\theta_{i}$ is the orientation angle of the measured ellipse.

The health degree $\Delta_{HD}$ is a derived metric from the health score $\Delta_{hs}$, designed to provide an intuitive assessment of the tunnel’s structural integrity. It is calculated as the reciprocal of one plus the health score, giving a value that ranges between 0 and 1. First, we calculated the $\Delta_{hs}$ by combining several normalized metrics that describe different aspects of the deformation, such as ΔC, $\Delta_{ab}$, and Δθ. Then, the $\Delta_{hs}$ is used to compute the $\Delta_{HD}$, which provides a more intuitive measure of the tunnel’s condition. Equation (9) is used to compute the health score.

(9) $$\Delta_{hs}=w_{c}\cdot \Delta C+w_{a}\cdot \Delta_{ab}+w_{\theta }\cdot \Delta \theta$$

where $w_{c}$, $w_{a}$, and $w_{\theta }$ are weights for the center displacement, axis length difference, and angle orientation difference, respectively. In the algorithm designed, $w_{c}$ = 1, $w_{a}$ = 1, and $w_{\theta }$ = 0.5.

Finally, the health degree is calculated using Equation (10).

(10) $$\Delta_{HD}=\frac{1}{1+\Delta_{hs}}$$

The health degree offers a straightforward interpretation: the closer the value is to 100%, the healthier the tunnel is considered to be. This measure is useful for summarizing complex deformation data into a single, easily understandable metric.

The radial deformation at an angle α is computed by comparing the radii, $R_{0}$ and $R_{i}$, of the ellipses at that angle (between the ellipse design and the ellipse measured). The radius of an ellipse at an angle α is given by Equation (11).

(11) $$\begin{array}{l} R_{0}=\frac{a_{0}\cdot b_{0}}{\sqrt{{\left(b_{0}\cdot \cos\left(\alpha -\theta_{0}\right)\right)}^{2}+{\left(a_{0}\cdot \sin\left(\alpha -\theta_{0}\right)\right)}^{2}}}\\ R_{i}=\frac{a_{i}\cdot b_{i}}{\sqrt{{\left(b_{i}\cdot \cos\left(\alpha -\theta_{i}\right)\right)}^{2}+{\left(a_{i}\cdot \sin\left(\alpha -\theta_{i}\right)\right)}^{2}}} \end{array}$$

where α is the angle for which the radii are being calculated.

Then, the radial deformation ΔR is calculated using Equation (12).

(12) $$\Delta R=R_{i}-R_{0}$$

**Figure 6:**
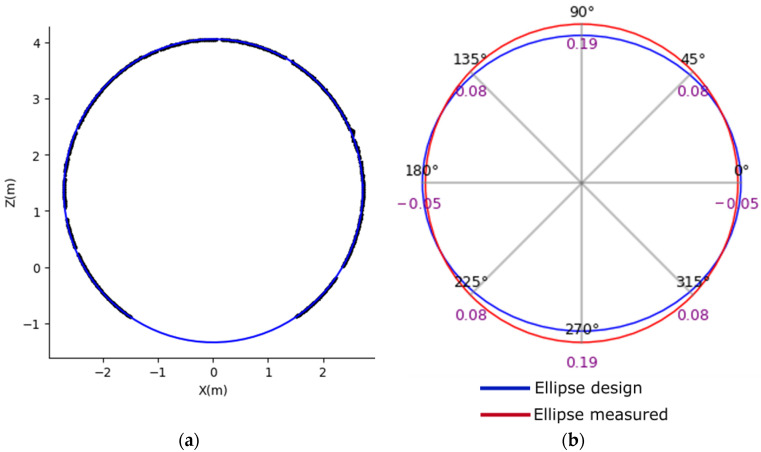
Tunnel cross-section radial deformation analysis. (**a**) Ellipse design fits to the shield tunnel point cloud. (**b**) Comparison between the ellipse fit from the point cloud measured and the ellipse design.

The algorithm gathers these metrics and calculates radial deformation at 360° equally spaced angles from 0 to 2π. Figure 6 illustrates the deformation detection using the radial deformation method between the reference and measured ellipses for the shield tunnel radial deformation analysis. The ellipse design, shown in blue, represents the shield ring’s original shape, while the ellipse measured, shown in red, depicts the current state. The differences between these ellipses are key indicators of the deformation experienced by the shield ring measured, providing a visual representation of changes in shape, size, and orientation. The grey lines show the radial directions used to calculate the deformation.

This methodology provided a systematic and detailed approach to monitoring the shield tunnels’ deformation. Figure 6 offers a comprehensive structural health assessment, identifying shields that may require attention and ensuring the tunnel’s long-term safety and functionality.

## 4. Method Validation

For this test, the metro tunnel on Line 2 in Guangzhou was measured. The tunnel is a circular shield structure with a designed inner diameter of 5.4 m and a ring width of 1.2 m. During the test, cross-section point cloud data were acquired using the proposed MLS system, as shown in Figure 1a. The experimental data covered the 104 rings, from shield rings 478 to 581, analyzing the difference between the measured and designed cross-sections by comparing typical sections over time. The individual shield rings were identified as research objects for deformation analysis and to define their health degree. Then, data measured using the Leica P16 system were used to validate the accuracy of the SILSS system by comparing the data from different measurements.

### 4.1. Data Preprocessing

**Figure 7:**
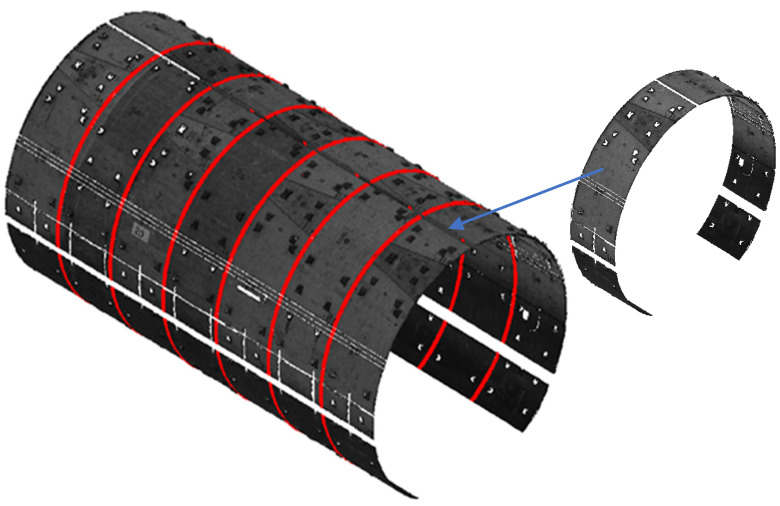
Individual shield ring separated, 3D views.

The circumferential joint was extracted based on the tunnel cross-section lining extraction result, and the tunnel structure lining point cloud was divided into individual shield rings. However, an algorithm for shield ring separation has been developed, relying on circumferential seam extraction. This approach was tested on 17 tunnel cross-sections containing 104 shield rings with a width of 1.2 m. Following the ring extraction algorithm execution, the shield rings were successfully obtained and considered as the research object for the shield tunnel deformation analysis, as illustrated in Figure 7.

### 4.2. Deformation Analysis

In this study, the experiment aimed to scan the same tunnel cross-sections using SILSS and validate the method with data from the Leica P16 system. Based on the data collected and parameters extracted in Section 3.2, the ellipticity change, center displacement, axis length changes, and health degree for 104 shield rings were calculated for deformation analysis and method validation. The radial deformation was measured for a cross-section at 360°.

#### 4.2.1. Ellipticity Change Precision Verification

The accuracy of the tunnel deformation monitoring system was validated by comparing its results to the ellipticity measured by the Leica system at the same location. The analysis results are presented in Table 3. The average absolute deviation between the ellipticity change obtained from the two devices was 0.19 mm, the maximum absolute deviation was 0.72 mm, and the minimum was 0.02 mm. According to the Technical Specification for Urban Rail Transit Engineering Monitoring [43], the accuracy requirement for detecting deformation in metro tunnels is ±3 mm. Therefore, the SILSS mobile laser scanning monitoring system can adequately fulfill the accuracy requirements for metro tunnel detection.

#### 4.2.2. Axis Length Change and Center Displacement

**Figure 8:**
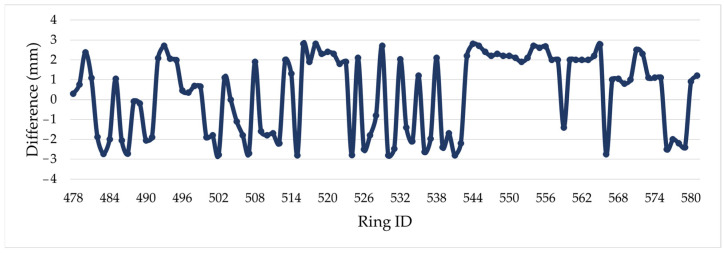
Difference in the axis length change: Comparison between the difference in axis length changes detected by the proposed method (SILSS) and the Leica P16.

**Figure 9:**
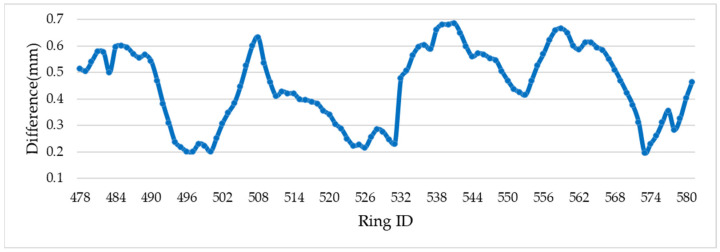
Difference in the center displacement. Comparison between the difference in ellipse center displacement detected by the proposed method (SILSS) and the Leica P16.

The axis length change and center displacement are calculated using point clouds t. By comparing the axis length change in the Leica P16 measurements(t + 1), it was found that the absolute deviation between the measurements was between 0 mm and 2.8 mm, and the average value was 1.89 mm. Shield rings for which the difference was less than 1 mm account for 11.54% of the data t + 1, those with a difference between 1 mm and 2 mm account for 39.42%, and those with a difference greater than 2 mm account for 49.04%, as shown in Figure 8. Then, the difference value of shield ring center displacement ranged from 0.20 to 0.68 mm and the average difference was 0.45 mm, as shown in Figure 9.

#### 4.2.3. Comparison of the Shield Ring Health Degree for Accuracy Verification

**Figure 10:**
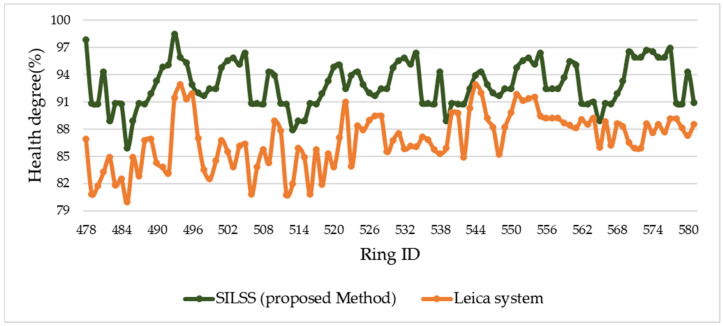
Shield ring health degree. Comparison between shield rings health degree detected by the proposed method (SILSS) and the Leica P16.

**Figure 11:**
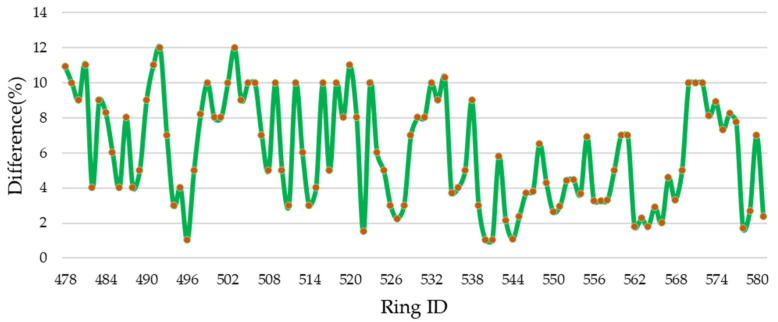
Difference in the shield ring health degree. Comparison between the difference in shield rings health degree detected by the proposed method (SILSS) and the Leica P16.

The shield ring health degree is defined as shown in Figure 10. Based on our measurement method, the health degree of the shield rings ranges between 86% and 98%. According to the Leica data, the health degree ranges between 80% and 93%. By comparing the shield ring health degree measurements, the differences ranged from 1% to 12%, as shown in Figure 11. Rings with a difference of less than 5% accounted for 49.04%, while those with a difference greater than 5% accounted for 50.96%.

The orange points represent the percentage difference measurement for each specific ring, indicating the precise value of the difference (%) at each ring. The green line connects these data points, highlighting the trend and variability of percentage differences across consecutive rings.

#### 4.2.4. Radial Deformation

**Figure 12:**
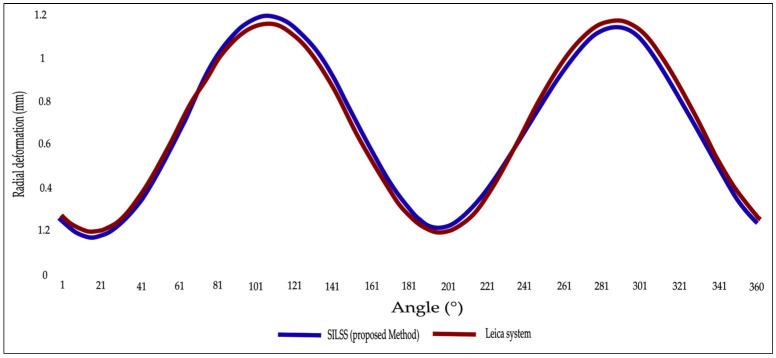
Radial deformation of the single tunnel cross-section. Comparison between the cross-section radial deformation detected by the proposed method (SILSS) and the Leica P16.

The radial deformation analysis for the cross-section is based on point cloud data measured with our system and validated using Leica P16 point cloud data at the same position. Deformation values were compared for a single cross-section at 360° from various angles, as shown in Figure 12. According to the comparison, the maximum radial deformation difference was 0.30 mm and the minimum difference was −0.02 mm.

## 5. Discussion

This study introduces a novel deformation analysis method based on mobile laser scanning (MLS), named SILSS. By incorporating point cloud preprocessing using the Random Sample Consensus (RANSAC) algorithm and tunnel cross-section deformation analysis, this approach presents significant advantages over traditional methods, such as manual inspections, total station measurements, and even widely used tools like Leica. The SILSS method offers faster data acquisition, broader coverage, and more precise quantitative analysis. Crucially, it enables a full cross-sectional deformation assessment, which is often lacking in other methods. The MLS technology allows for rapid, repeated data collection at different time intervals, ensuring consistent and precise deformation monitoring. Validation was carried out using a second dataset collected with the Leica P16 system in the same test area. The robust preprocessing and extraction of tunnel cross-section linings, circumferential seams, and individual shield rings significantly reduced the complexity of data processing. The use of ellipse fitting for 104 shield rings enabled detailed evaluation of ellipticity parameters, capturing deformation through key metrics such as ellipticity change, axis length changes, center displacement, health degree, and radial deformation.

This paper used five deformation analysis parameters to verify the precision of this method: absolute deviation of ellipticity change, center displacement, axis length changes, radial deformation, and health degree difference. When comparing the Leica P16 data with those obtained from SILSS, the results showed high accuracy. The average absolute deviation of ellipticity change was 0.19 mm, the axis length change was 1.89 mm, and the center displacement difference was 0.45 mm. Radial deformation showed an average difference of 0.64 mm. These results, with maximum deviations below 3 mm, meet the precision requirements for tunnel deformation monitoring [43]. The health degree of shield rings ranged from 86% to 98%, with a maximum difference of 12% and a minimum of 1%. According to the standards for tunnel engineering, a health degree above 80% is crucial for operational safety [44], which indicates that this method is better. Thus, the overall stability of the tunnel structure was good, and the deformation was within a small range.

Compared with existing studies, Yi et al. [45] reported that deformations exceeding 10 mm pose security risks, while Wan et al. [46] showed that MLS enhances convergence analysis and displacement tracking. Liu et al. [47] further emphasized the precision of MLS for comprehensive deformation assessments without physical targets. These studies support the findings of this paper, which confirm the reliability and precision of the SILSS method, especially with the integration of the RANSAC algorithm to minimize errors [21,48].

Despite the method’s advantages, challenges remain. The method relies heavily on the availability of shield ring design data and the quality of the point cloud. Additionally, it is currently limited to circular shield tunnels. Future work could focus on expanding the applicability of the method to tunnels with different shapes, such as rectangular or irregular profiles. One promising direction would be the integration of deep learning techniques to adapt the deformation analysis to various tunnel geometries. Moreover, algorithmic improvements, such as enhancing RANSAC-based preprocessing for more complex tunnel environments, could further increase the robustness of this approach.

## 6. Conclusions

This study presents a novel approach for analyzing shield tunnel deformation using raw point cloud data collected through the Self-mobile Intelligent Laser Scanning System (SILSS). By focusing on tunnel cross-section point cloud lining extraction and ellipse fitting, this method significantly enhances the accuracy of tunnel deformation monitoring over time. The effectiveness of the approach has been demonstrated in the context of structural health monitoring. The key findings are summarized as follows:

1. This study highlighted the effectiveness of MLS point cloud preprocessing for cross-sectional tunnel monitoring. Using the Random Sample Consensus (RANSAC) algorithm for ellipse fitting, meaningful tunnel cross-section linings were successfully extracted. Additional steps involved the separation of individual shield rings and the extraction of key deformation parameters (as shown in Table 1 and Table 2). This approach enabled the evaluation of 104 shield rings across 17 cross-sections, and 5 critical deformation parameters were used for analysis. Validation of the method was performed using tunnel cross-section point clouds obtained from the Leica P16 system.
2. The experimental results demonstrated that the precision of deformation analysis was within 3 mm. The comparison of key deformation metrics between SILSS and the Leica P16 system showed differences of less than 3 mm, further validating the method. Additionally, the shield ring health degree was defined based on this comparison, with health degree differences ranging from 1% to 12%. These values align with industry standards, confirming the accuracy of the data collected by the SILSS. The qualitative assessment of shield tunnel deformation, based on point clouds collected at two time periods, demonstrated that the differences fell within an acceptable range, ensuring the continued safety and stability of the tunnel.

## Figures and Tables

**Table 1:** Parameters extracted from the SILSS point cloud dataset by shield ring.

Ring ID	Ellipticity	Semi-Major Axis a (m)	Semi-Minor Axis b (m)	Center, cx (m)	Center, cy (m)	Rotation Angle, Theta (°)
478	0.122	2.712	2.691	0.153	1.324	0.027
479	0.123	2.712	2.691	0.154	1.330	3.100
480	0.110	2.710	2.693	0.153	1.337	3.082
481	0.106	2.709	2.694	0.151	1.344	0.018
482	0.102	2.709	2.694	0.150	1.350	0.027
483	0.083	2.707	2.697	0.146	1.360	0.638
484	0.075	2.707	2.699	0.144	1.362	0.895
485	0.075	2.707	2.699	0.141	1.358	1.144
486	0.100	2.709	2.695	0.141	1.359	0.456
487	0.112	2.710	2.693	0.140	1.358	0.31
488	0.134	2.715	2.690	0.139	1.353	0.32
489	0.118	2.711	2.692	0.134	1.353	0.248
490	0.123	2.711	2.691	0.129	1.355	0.157
491	0.129	2.713	2.690	0.126	1.354	0.053
492	0.122	2.712	2.692	0.123	1.354	0.039
493	0.110	2.710	2.693	0.121	1.358	0.227
494	0.100	2.708	2.695	0.120	1.356	0.122
495	0.088	2.707	2.697	0.114	1.357	0.101
496	0.099	2.708	2.694	0.111	1.360	0.212
497	0.088	2.706	2.696	0.105	1.355	0.293
498	0.081	2.706	2.697	0.104	1.355	0.314
499	0.079	2.706	2.698	0.100	1.356	0.262
500	0.092	2.707	2.696	0.095	1.357	3.141
501	0.091	2.708	2.697	0.094	1.356	0.113
502	0.091	2.707	2.696	0.095	1.352	0.064

**Table 2:** Parameters extracted from Leica P16 point cloud dataset by shield ring.

Ring ID	Ellipticity	Semi-Major Axis a (m)	Semi-Minor Axis b (m)	Center, cx (m)	Center, cy (m)	Rotation Angle, Theta (°)
478	0.118	2.712	2.693	0.068	1.324	3.020
479	0.122	2.712	2.692	0.067	1.328	3.047
480	0.111	2.711	2.694	0.068	1.335	3.061
481	0.107	2.710	2.694	0.067	1.342	3.011
482	0.106	2.709	2.694	0.064	1.347	2.984
483	0.095	2.709	2.697	0.064	1.355	2.671
484	0.090	2.708	2.697	0.028	1.357	2.850
485	0.103	2.708	2.694	0.026	1.359	2.806
486	0.097	2.708	2.695	0.021	1.356	2.701
487	0.092	2.707	2.695	0.019	1.355	2.664
488	0.089	2.707	2.696	0.016	1.356	2.642
489	0.089	2.707	2.696	0.010	1.356	2.903
490	0.096	2.708	2.696	0.007	1.354	2.905
491	0.102	2.709	2.695	0.009	1.348	2.970
492	0.119	2.712	2.693	0.006	1.338	2.918
493	0.131	2.714	2.691	0.008	1.329	2.973
494	0.126	2.713	2.692	0.010	1.320	3.040
495	0.120	2.712	2.693	0.015	1.313	3.048
496	0.117	2.711	2.693	0.017	1.302	3.060
497	0.113	2.710	2.693	0.016	1.294	3.126
498	0.117	2.711	2.693	0.018	1.288	3.062
499	0.113	2.710	2.693	0.018	1.290	3.083
500	0.112	2.710	2.693	0.017	1.299	3.049
501	0.105	2.709	2.694	0.022	1.309	3.107
502	0.100	2.708	2.695	0.022	1.320	3.074

**Table 3:** Comparison of ellipticity change measured by SILSS and by Leica P16.

Ring ID	SILSS (Method Proposed) (mm)	Leica P16 Method (mm)	Absolute Deviation (mm)
478	0.06	0.58	0.53
479	0.16	0.41	0.25
480	0.20	0.24	0.04
481	0.32	0.52	0.21
482	0.22	0.40	0.18
483	0.15	0.31	0.17
484	0.43	0.59	0.16
485	0.21	0.50	0.29
486	0.17	0.31	0.14
487	0.22	0.94	0.72
488	0.11	0.41	0.30
489	0.25	0.83	0.58
490	0.46	0.61	0.15
491	0.31	0.55	0.24
492	0.33	0.30	0.03
493	0.14	0.30	0.16
494	0.24	0.41	0.18
495	0.13	0.69	0.56
496	0.23	0.51	0.29
497	0.32	0.51	0.18
498	0.53	0.59	0.07
499	0.22	0.38	0.16
500	0.22	0.41	0.19
501	0.32	0.51	0.19
502	0.31	0.42	0.11

## Data Availability

Data can be obtained from the authors upon request.
